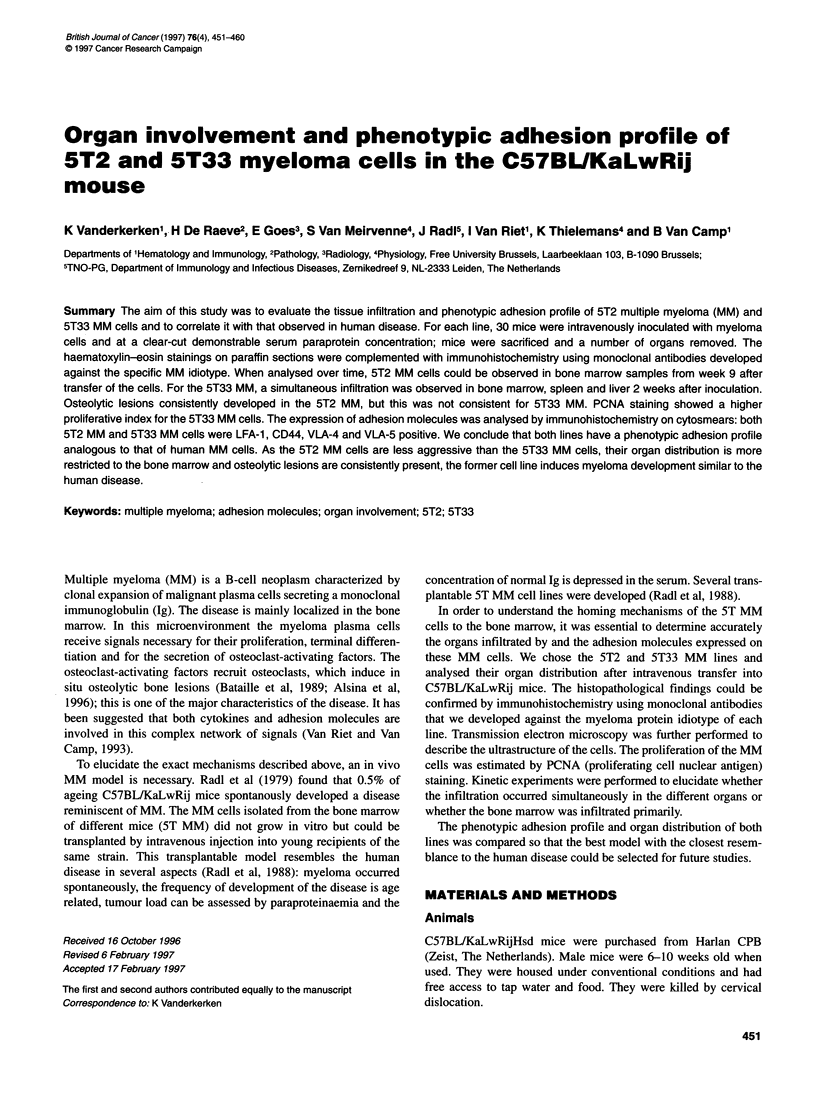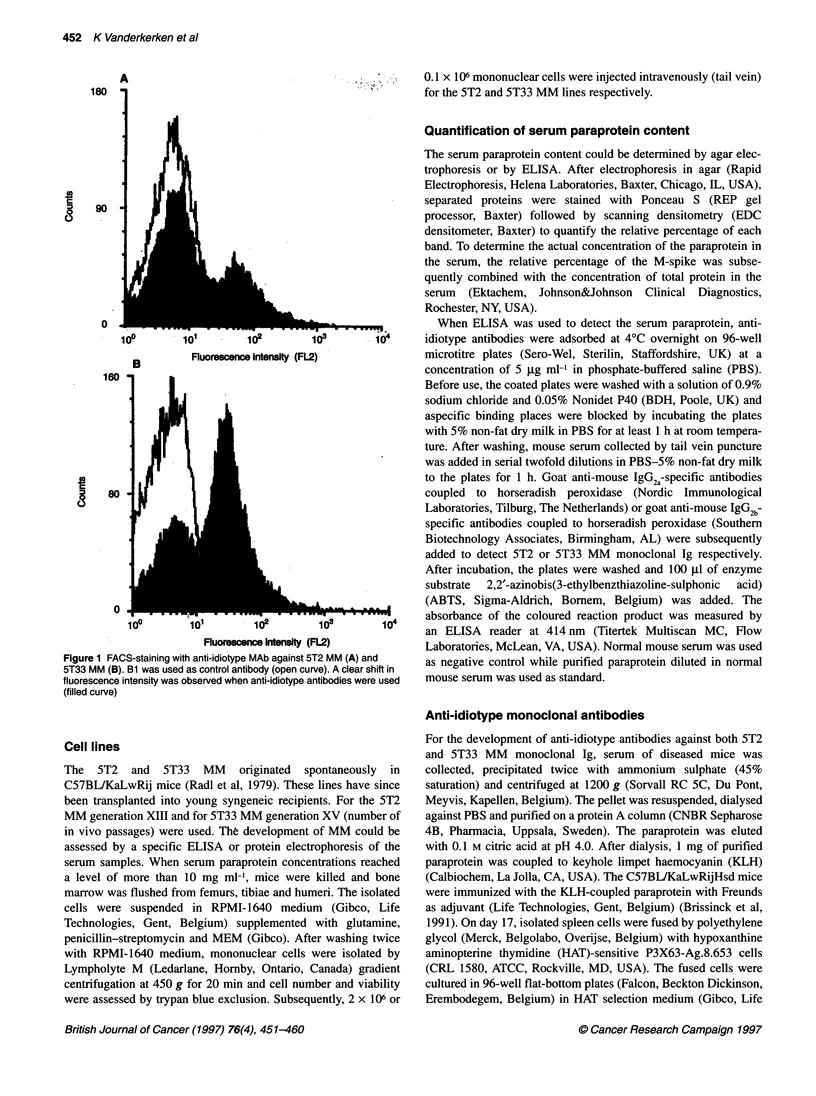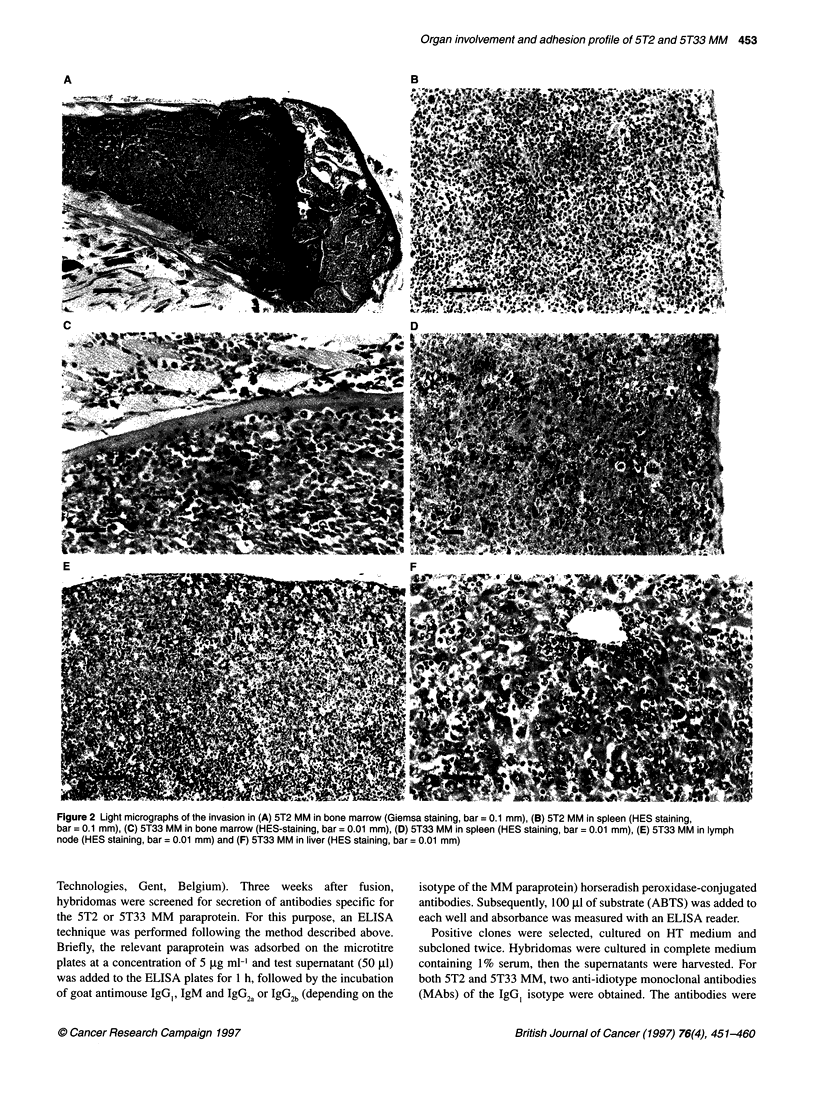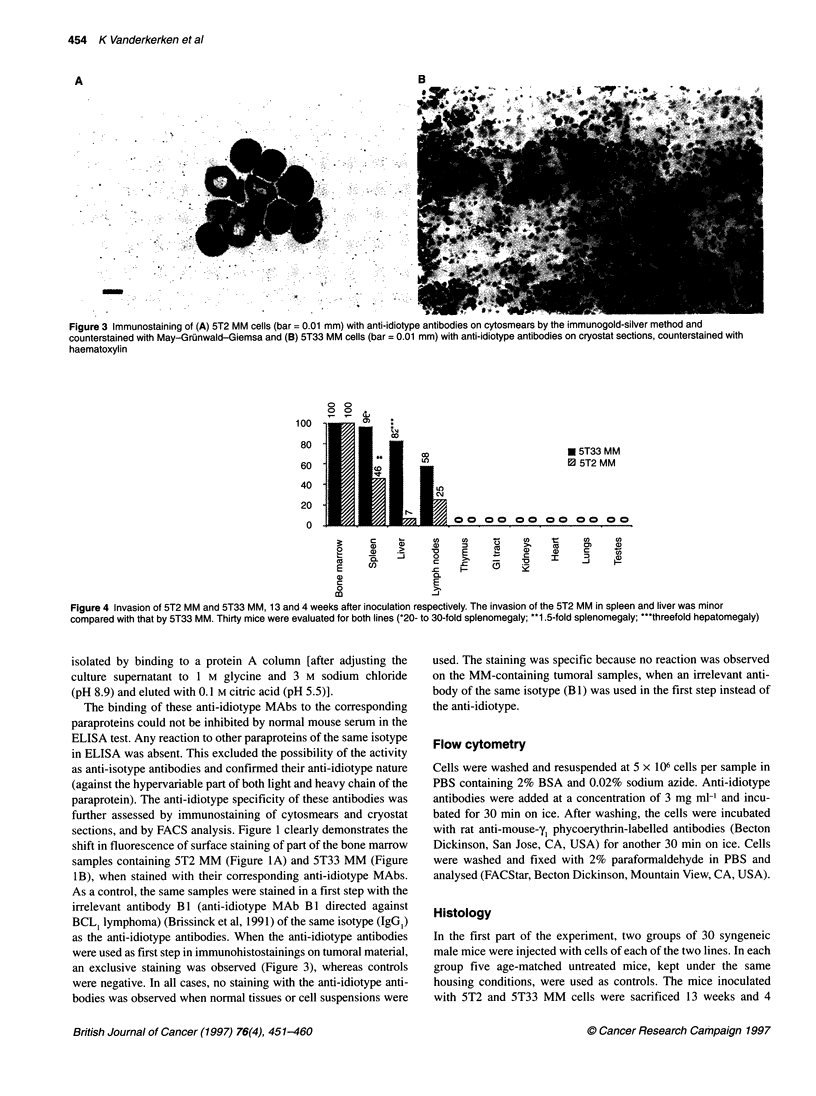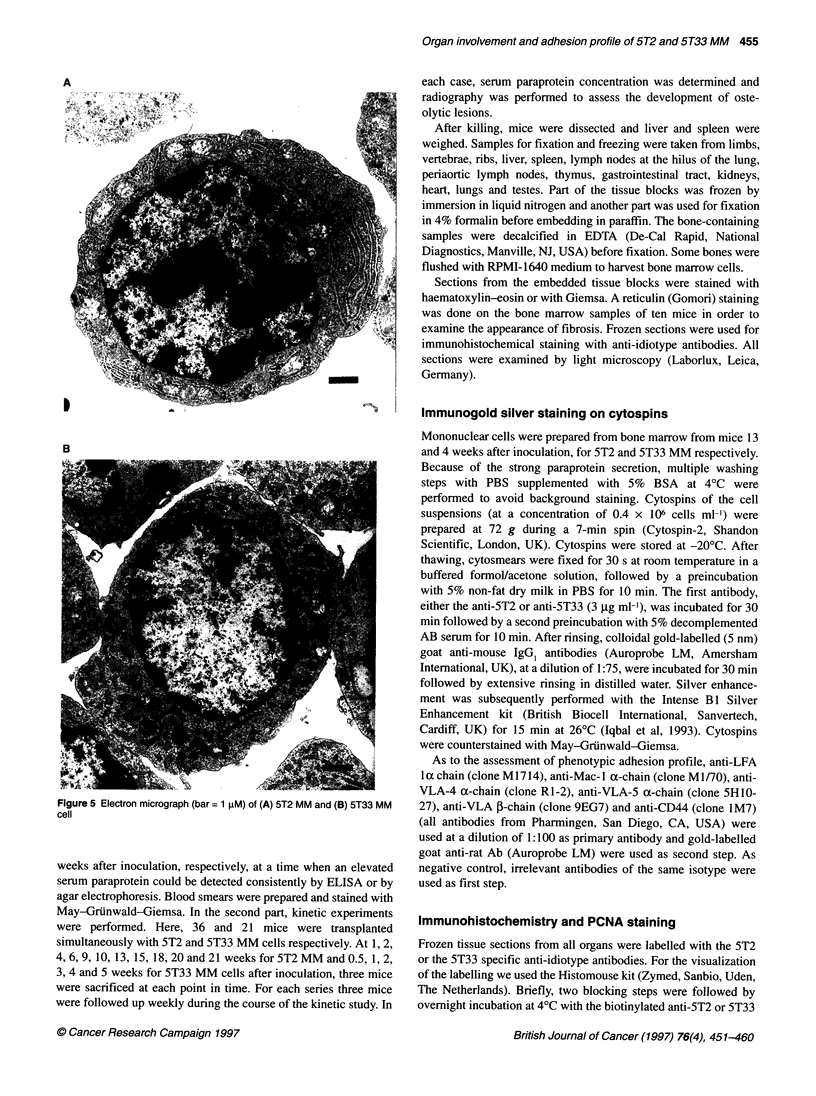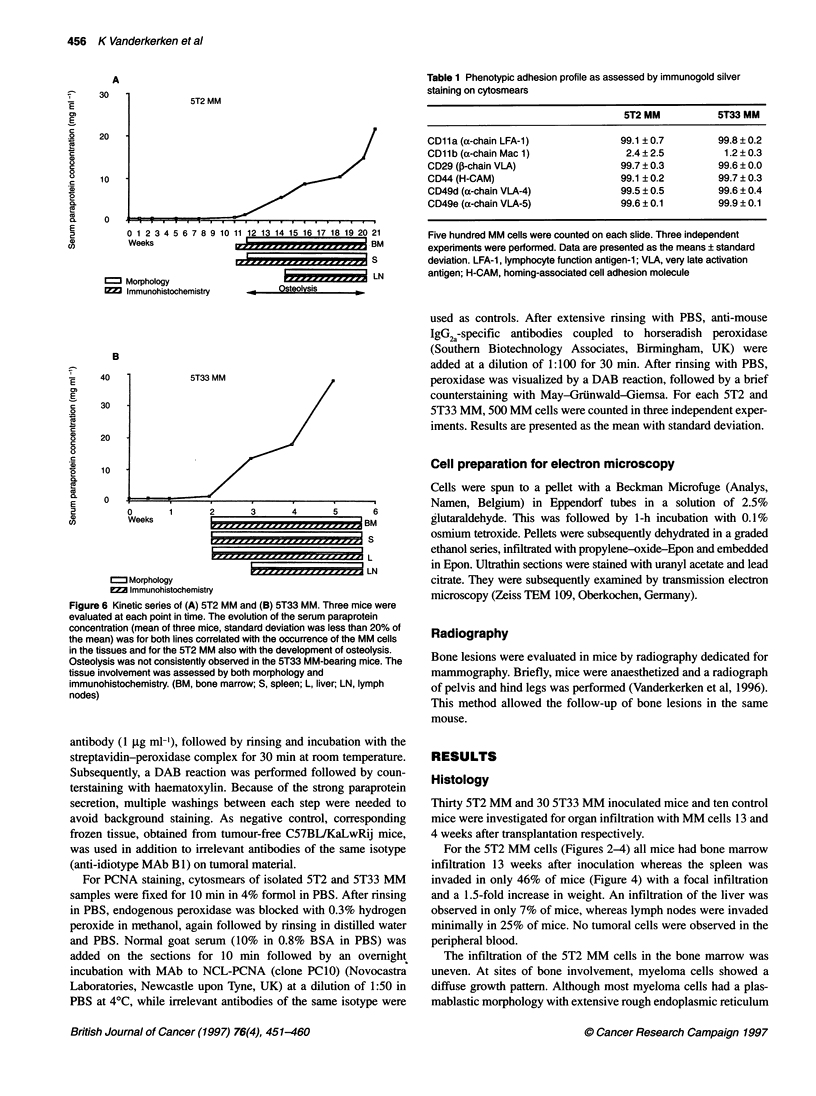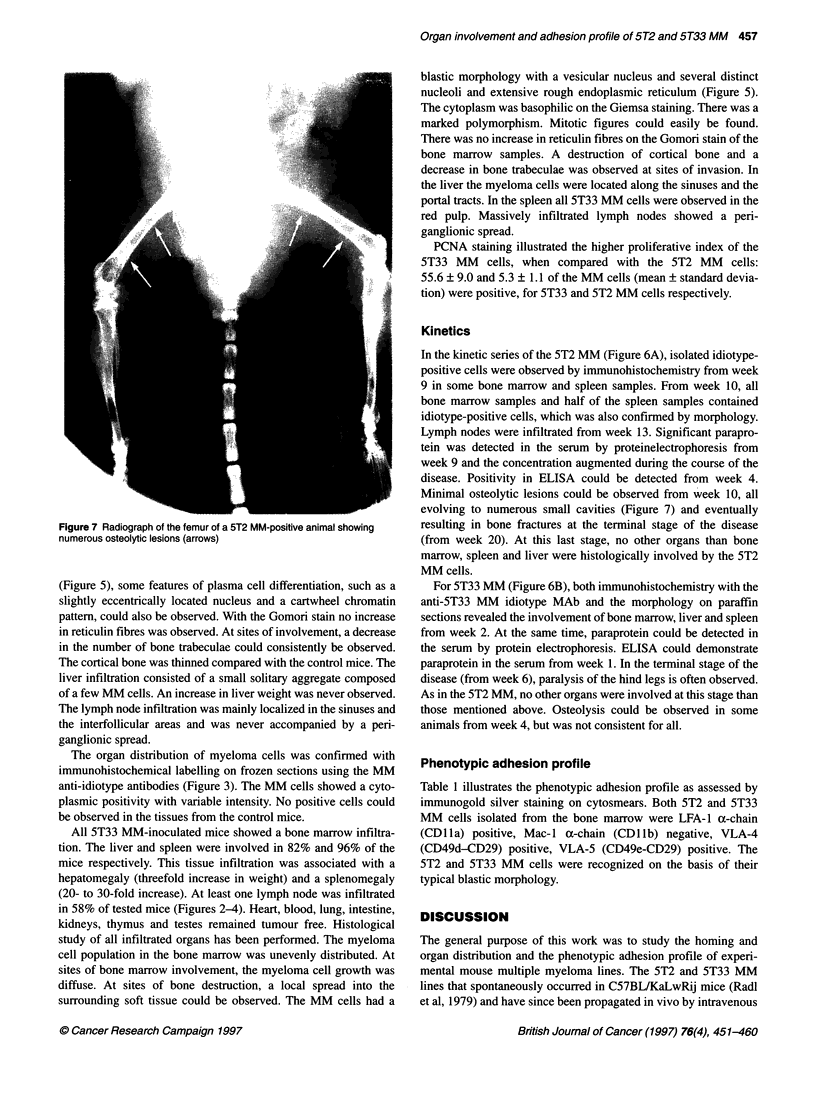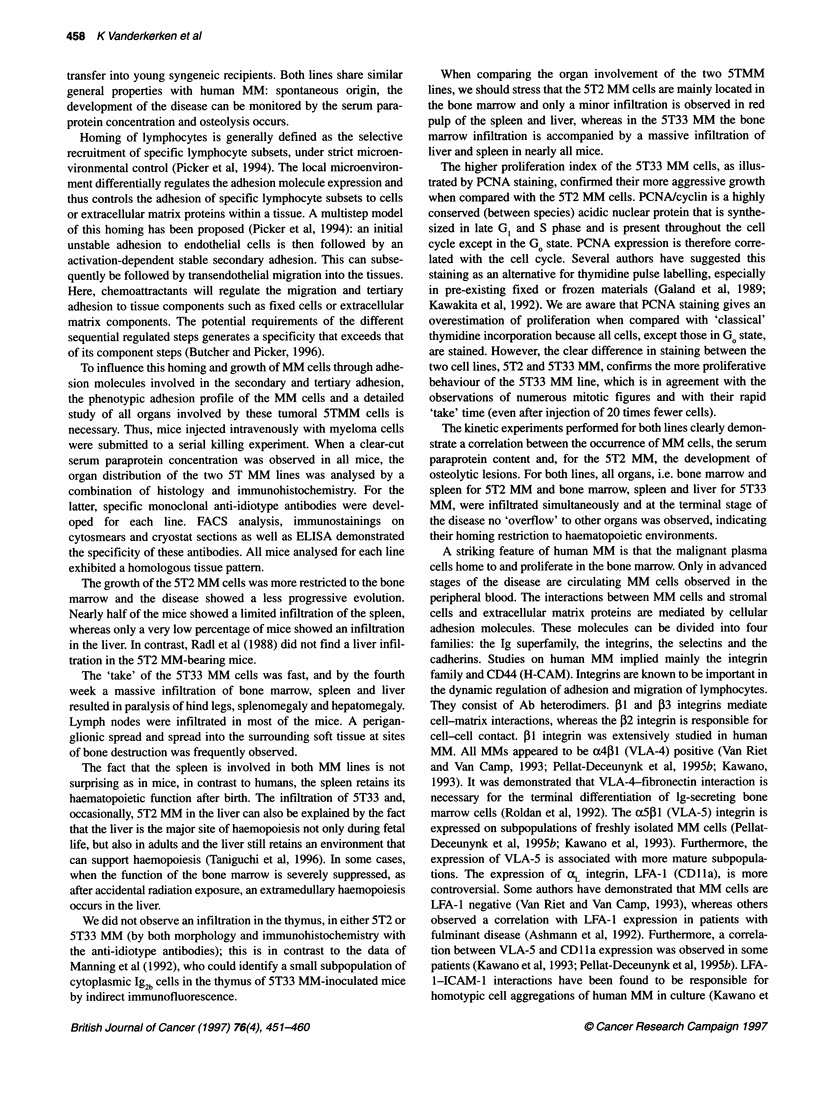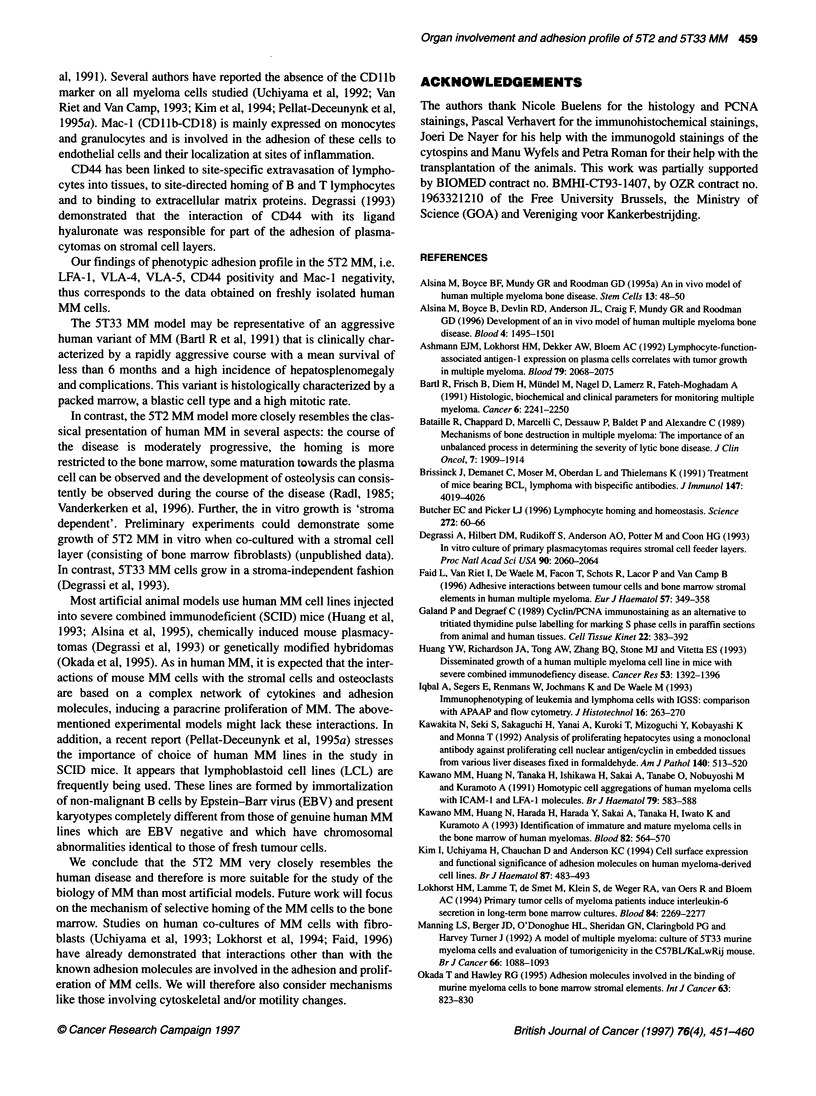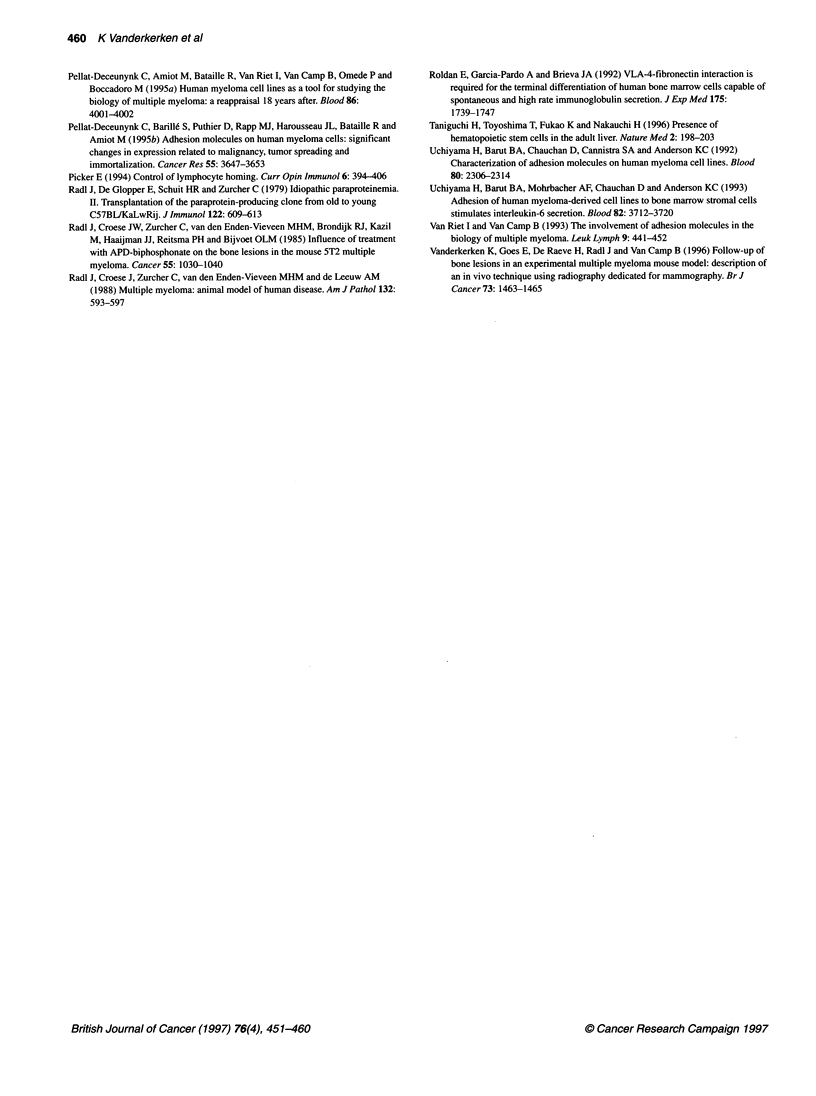# Organ involvement and phenotypic adhesion profile of 5T2 and 5T33 myeloma cells in the C57BL/KaLwRij mouse.

**DOI:** 10.1038/bjc.1997.409

**Published:** 1997

**Authors:** K. Vanderkerken, H. De Raeve, E. Goes, S. Van Meirvenne, J. Radl, I. Van Riet, K. Thielemans, B. Van Camp

**Affiliations:** Department of Hematology and Immunology, Free University Brussels.

## Abstract

**Images:**


British Journal of Cancer (1997) 76(4), 451–460
© 1997 Cancer Research Campaign

### Organ involvement and phenotypic adhesion profile of 5T2 and 5T33 myeloma cells in the C57BL/KaLwRij mouse

**K Vanderkerken[1], H De Raeve[2], E Goes[3], S Van Meirvenne[4], J Radl[5], I Van Riet[1], K Thielemans[4] and B Van Camp[1]**

Departments of [1]Hematology and Immunology, [2]Pathology, [3]Radiology, [4]Physiology, Free University Brussels, Laarbeeklaan 103, B-1090 Brussels; [5]TNO-PG, Department of Immunology and Infectious Diseases, Zernikedreef 9, NL-2333 Leiden, The Netherlands

**Summary** The aim of this study was to evaluate the tissue infiltration and phenotypic adhesion profile of 5T2 multiple myeloma (MM) and 5T33 MM cells and to correlate it with that observed in human disease. For each line, 30 mice were intravenously inoculated with myeloma cells and at a clear-cut demonstrable serum paraprotein concentration; mice were sacrificed and a number of organs removed. The haematoxylin–eosin stainings on paraffin sections were complemented with immunohistochemistry using monoclonal antibodies developed against the specific MM idiotype. When analysed over time, 5T2 MM cells could be observed in bone marrow samples from week 9 after transfer of the cells. For the 5T33 MM, a simultaneous infiltration was observed in bone marrow, spleen and liver 2 weeks after inoculation. Osteolytic lesions consistently developed in the 5T2 MM, but this was not consistent for 5T33 MM. PCNA staining showed a higher proliferative index for the 5T33 MM cells. The expression of adhesion molecules was analysed by immunohistochemistry on cytosmears: both 5T2 MM and 5T33 MM cells were LFA-1, CD44, VLA-4 and VLA-5 positive. We conclude that both lines have a phenotypic adhesion profile analogous to that of human MM cells. As the 5T2 MM cells are less aggressive than the 5T33 MM cells, their organ distribution is more restricted to the bone marrow and osteolytic lesions are consistently present, the former cell line induces myeloma development similar to the human disease.

**Keywords:** multiple myeloma; adhesion molecules; organ involvement; 5T2; 5T33

Multiple myeloma (MM) is a B-cell neoplasm characterized by clonal expansion of malignant plasma cells secreting a monoclonal immunoglobulin (Ig). The disease is mainly localized in the bone marrow. In this microenvironment the myeloma plasma cells receive signals necessary for their proliferation, terminal differentiation and for the secretion of osteoclast-activating factors. The osteoclast-activating factors recruit osteoclasts, which induce in situ osteolytic bone lesions (Bataille et al, 1989; Alsina et al, 1996); this is one of the major characteristics of the disease. It has been suggested that both cytokines and adhesion molecules are involved in this complex network of signals (Van Riet and Van Camp, 1993).

To elucidate the exact mechanisms described above, an in vivo MM model is necessary. Radl et al (1979) found that 0.5% of ageing C57BL/KaLwRij mice spontanously developed a disease reminiscent of MM. The MM cells isolated from the bone marrow of different mice (5T MM) did not grow in vitro but could be transplanted by intravenous injection into young recipients of the same strain. This transplantable model resembles the human disease in several aspects (Radl et al, 1988): myeloma occurred spontaneously, the frequency of development of the disease is age related, tumour load can be assessed by paraproteinaemia and the concentration of normal Ig is depressed in the serum. Several transplantable 5T MM cell lines were developed (Radl et al, 1988).

In order to understand the homing mechanisms of the 5T MM cells to the bone marrow, it was essential to determine accurately the organs infiltrated by and the adhesion molecules expressed on these MM cells. We chose the 5T2 and 5T33 MM lines and analysed their organ distribution after intravenous transfer into C57BL/KaLwRij mice. The histopathological findings could be confirmed by immunohistochemistry using monoclonal antibodies that we developed against the myeloma protein idiotype of each line. Transmission electron microscopy was further performed to describe the ultrastructure of the cells. The proliferation of the MM cells was estimated by PCNA (proliferating cell nuclear antigen) staining. Kinetic experiments were performed to elucidate whether the infiltration occurred simultaneously in the different organs or whether the bone marrow was infiltrated primarily.

The phenotypic adhesion profile and organ distribution of both lines was compared so that the best model with the closest resemblance to the human disease could be selected for future studies.

### MATERIALS AND METHODS

### Animals

C57BL/KaLwRijHsd mice were purchased from Harlan CPB (Zeist, The Netherlands). Male mice were 6–10 weeks old when used. They were housed under conventional conditions and had free access to tap water and food. They were killed by cervical dislocation.

*Received 16 October 1996*
*Revised 6 February 1997*
*Accepted 17 February 1997*

The first and second authors contributed equally to the manuscript
*Correspondence to:* K Vanderkerken

**Figure 1** FACS-staining with anti-idiotype MAb against 5T2 MM (**A**) and 5T33 MM (**B**). B1 was used as control antibody (open curve). A clear shift in fluorescence intensity was observed when anti-idiotype antibodies were used (filled curve)

### Cell lines

The 5T2 and 5T33 MM originated spontaneously in C57BL/KaLwRij mice (Radl et al, 1979). These lines have since been transplanted into young syngeneic recipients. For the 5T2 MM generation XIII and for 5T33 MM generation XV (number of in vivo passages) were used. The development of MM could be assessed by a specific ELISA or protein electrophoresis of the serum samples. When serum paraprotein concentrations reached a level of more than 10 mg ml$^{-1}$, mice were killed and bone marrow was flushed from femurs, tibiae and humeri. The isolated cells were suspended in RPMI-1640 medium (Gibco, Life Technologies, Gent, Belgium) supplemented with glutamine, penicillin–streptomycin and MEM (Gibco). After washing twice with RPMI-1640 medium, mononuclear cells were isolated by Lympholyte M (Ledarlane, Hornby, Ontario, Canada) gradient centrifugation at 450 ***g*** for 20 min and cell number and viability were assessed by trypan blue exclusion. Subsequently, $2 \times 10^6$ or $0.1 \times 10^6$ mononuclear cells were injected intravenously (tail vein) for the 5T2 and 5T33 MM lines respectively.

### Quantification of serum paraprotein content

The serum paraprotein content could be determined by agar electrophoresis or by ELISA. After electrophoresis in agar (Rapid Electrophoresis, Helena Laboratories, Baxter, Chicago, IL, USA), separated proteins were stained with Ponceau S (REP gel processor, Baxter) followed by scanning densitometry (EDC densitometer, Baxter) to quantify the relative percentage of each band. To determine the actual concentration of the paraprotein in the serum, the relative percentage of the M-spike was subsequently combined with the concentration of total protein in the serum (Ektachem, Johnson&Johnson Clinical Diagnostics, Rochester, NY, USA).

When ELISA was used to detect the serum paraprotein, anti-idiotype antibodies were adsorbed at 4°C overnight on 96-well microtitre plates (Sero-Wel, Sterilin, Staffordshire, UK) at a concentration of 5 µg ml$^{-1}$ in phosphate-buffered saline (PBS). Before use, the coated plates were washed with a solution of 0.9% sodium chloride and 0.05% Nonidet P40 (BDH, Poole, UK) and aspecific binding places were blocked by incubating the plates with 5% non-fat dry milk in PBS for at least 1 h at room temperature. After washing, mouse serum collected by tail vein puncture was added in serial twofold dilutions in PBS–5% non-fat dry milk to the plates for 1 h. Goat anti-mouse $IgG_{2a}$-specific antibodies coupled to horseradish peroxidase (Nordic Immunological Laboratories, Tilburg, The Netherlands) or goat anti-mouse $IgG_{2b}$-specific antibodies coupled to horseradish peroxidase (Southern Biotechnology Associates, Birmingham, AL) were subsequently added to detect 5T2 or 5T33 MM monoclonal Ig respectively. After incubation, the plates were washed and 100 µl of enzyme substrate 2,2′-azinobis(3-ethylbenzthiazoline-sulphonic acid) (ABTS, Sigma-Aldrich, Bornem, Belgium) was added. The absorbance of the coloured reaction product was measured by an ELISA reader at 414 nm (Titertek Multiscan MC, Flow Laboratories, McLean, VA, USA). Normal mouse serum was used as negative control while purified paraprotein diluted in normal mouse serum was used as standard.

### Anti-idiotype monoclonal antibodies

For the development of anti-idiotype antibodies against both 5T2 and 5T33 MM monoclonal Ig, serum of diseased mice was collected, precipitated twice with ammonium sulphate (45% saturation) and centrifuged at 1200 ***g*** (Sorvall RC 5C, Du Pont, Meyvis, Kapellen, Belgium). The pellet was resuspended, dialysed against PBS and purified on a protein A column (CNBR Sepharose 4B, Pharmacia, Uppsala, Sweden). The paraprotein was eluted with 0.1 M citric acid at pH 4.0. After dialysis, 1 mg of purified paraprotein was coupled to keyhole limpet haemocyanin (KLH) (Calbiochem, La Jolla, CA, USA). The C57BL/KaLwRijHsd mice were immunized with the KLH-coupled paraprotein with Freunds as adjuvant (Life Technologies, Gent, Belgium) (Brissinck et al, 1991). On day 17, isolated spleen cells were fused by polyethylene glycol (Merck, Belgolabo, Overijse, Belgium) with hypoxanthine aminopterine thymidine (HAT)-sensitive P3X63-Ag.8.653 cells (CRL 1580, ATCC, Rockville, MD, USA). The fused cells were cultured in 96-well flat-bottom plates (Falcon, Beckton Dickinson, Erembodegem, Belgium) in HAT selection medium (Gibco, Life

© Cancer Research Campaign 1997

**Figure 2** Light micrographs of the invasion in (**A**) 5T2 MM in bone marrow (Giemsa staining, bar = 0.1 mm), (**B**) 5T2 MM in spleen (HES staining, bar = 0.1 mm), (**C**) 5T33 MM in bone marrow (HES-staining, bar = 0.01 mm), (**D**) 5T33 MM in spleen (HES staining, bar = 0.01 mm), (**E**) 5T33 MM in lymph node (HES staining, bar = 0.01 mm) and (**F**) 5T33 MM in liver (HES staining, bar = 0.01 mm)

Technologies, Gent, Belgium). Three weeks after fusion, hybridomas were screened for secretion of antibodies specific for the 5T2 or 5T33 MM paraprotein. For this purpose, an ELISA technique was performed following the method described above. Briefly, the relevant paraprotein was adsorbed on the microtitre plates at a concentration of 5 μg $ml^{-1}$ and test supernatant (50 μl) was added to the ELISA plates for 1 h, followed by the incubation of goat antimouse $IgG_1$, IgM and $IgG_{2a}$ or $IgG_{2b}$ (depending on the isotype of the MM paraprotein) horseradish peroxidase-conjugated antibodies. Subsequently, 100 μl of substrate (ABTS) was added to each well and absorbance was measured with an ELISA reader.

Positive clones were selected, cultured on HT medium and subcloned twice. Hybridomas were cultured in complete medium containing 1% serum, then the supernatants were harvested. For both 5T2 and 5T33 MM, two anti-idiotype monoclonal antibodies (MAbs) of the $IgG_1$ isotype were obtained. The antibodies were

© Cancer Research Campaign 1997

**Figure 3** Immunostaining of (**A**) 5T2 MM cells (bar = 0.01 mm) with anti-idiotype antibodies on cytosmears by the immunogold-silver method and counterstained with May–Grünwald–Giemsa and (**B**) 5T33 MM cells (bar = 0.01 mm) with anti-idiotype antibodies on cryostat sections, counterstained with haematoxylin

**Figure 4** Invasion of 5T2 MM and 5T33 MM, 13 and 4 weeks after inoculation respectively. The invasion of the 5T2 MM in spleen and liver was minor compared with that by 5T33 MM. Thirty mice were evaluated for both lines (*20- to 30-fold splenomegaly; **1.5-fold splenomegaly; ***threefold hepatomegaly)

isolated by binding to a protein A column [after adjusting the culture supernatant to 1 M glycine and 3 M sodium chloride (pH 8.9) and eluted with 0.1 M citric acid (pH 5.5)].

The binding of these anti-idiotype MAbs to the corresponding paraproteins could not be inhibited by normal mouse serum in the ELISA test. Any reaction to other paraproteins of the same isotype in ELISA was absent. This excluded the possibility of the activity as anti-isotype antibodies and confirmed their anti-idiotype nature (against the hypervariable part of both light and heavy chain of the paraprotein). The anti-idiotype specificity of these antibodies was further assessed by immunostaining of cytosmears and cryostat sections, and by FACS analysis. Figure 1 clearly demonstrates the shift in fluorescence of surface staining of part of the bone marrow samples containing 5T2 MM (Figure 1A) and 5T33 MM (Figure 1B), when stained with their corresponding anti-idiotype MAbs. As a control, the same samples were stained in a first step with the irrelevant antibody B1 (anti-idiotype MAb B1 directed against $BCL_1$ lymphoma) (Brissinck et al, 1991) of the same isotype ($IgG_1$) as the anti-idiotype antibodies. When the anti-idiotype antibodies were used as first step in immunohistostainings on tumoral material, an exclusive staining was observed (Figure 3), whereas controls were negative. In all cases, no staining with the anti-idiotype antibodies was observed when normal tissues or cell suspensions were used. The staining was specific because no reaction was observed on the MM-containing tumoral samples, when an irrelevant antibody of the same isotype (B1) was used in the first step instead of the anti-idiotype.

### Flow cytometry

Cells were washed and resuspended at $5 \times 10^6$ cells per sample in PBS containing 2% BSA and 0.02% sodium azide. Anti-idiotype antibodies were added at a concentration of 3 mg $ml^{-1}$ and incubated for 30 min on ice. After washing, the cells were incubated with rat anti-mouse-$\gamma_1$ phycoerythrin-labelled antibodies (Becton Dickinson, San Jose, CA, USA) for another 30 min on ice. Cells were washed and fixed with 2% paraformaldehyde in PBS and analysed (FACStar, Becton Dickinson, Mountain View, CA, USA).

### Histology

In the first part of the experiment, two groups of 30 syngeneic male mice were injected with cells of each of the two lines. In each group five age-matched untreated mice, kept under the same housing conditions, were used as controls. The mice inoculated with 5T2 and 5T33 MM cells were sacrificed 13 weeks and 4

© Cancer Research Campaign 1997

A

B

**Figure 5** Electron micrograph (bar = 1 μM) of (**A**) 5T2 MM and (**B**) 5T33 MM cell weeks after inoculation, respectively, at a time when an elevated serum paraprotein could be detected consistently by ELISA or by agar electrophoresis. Blood smears were prepared and stained with May–Grünwald–Giemsa. In the second part, kinetic experiments were performed. Here, 36 and 21 mice were transplanted simultaneously with 5T2 and 5T33 MM cells respectively. At 1, 2, 4, 6, 9, 10, 13, 15, 18, 20 and 21 weeks for 5T2 MM and 0.5, 1, 2, 3, 4 and 5 weeks for 5T33 MM cells after inoculation, three mice were sacrificed at each point in time. For each series three mice were followed up weekly during the course of the kinetic study. In each case, serum paraprotein concentration was determined and radiography was performed to assess the development of osteolytic lesions.

After killing, mice were dissected and liver and spleen were weighed. Samples for fixation and freezing were taken from limbs, vertebrae, ribs, liver, spleen, lymph nodes at the hilus of the lung, periaortic lymph nodes, thymus, gastrointestinal tract, kidneys, heart, lungs and testes. Part of the tissue blocks was frozen by immersion in liquid nitrogen and another part was used for fixation in 4% formalin before embedding in paraffin. The bone-containing samples were decalcified in EDTA (De-Cal Rapid, National Diagnostics, Manville, NJ, USA) before fixation. Some bones were flushed with RPMI-1640 medium to harvest bone marrow cells.

Sections from the embedded tissue blocks were stained with haematoxylin–eosin or with Giemsa. A reticulin (Gomori) staining was done on the bone marrow samples of ten mice in order to examine the appearance of fibrosis. Frozen sections were used for immunohistochemical staining with anti-idiotype antibodies. All sections were examined by light microscopy (Laborlux, Leica, Germany).

### Immunogold silver staining on cytospins

Mononuclear cells were prepared from bone marrow from mice 13 and 4 weeks after inoculation, for 5T2 and 5T33 MM respectively. Because of the strong paraprotein secretion, multiple washing steps with PBS supplemented with 5% BSA at 4°C were performed to avoid background staining. Cytospins of the cell suspensions (at a concentration of $0.4 \times 10^6$ cells $ml^{-1}$) were prepared at 72 *g* during a 7-min spin (Cytospin-2, Shandon Scientific, London, UK). Cytospins were stored at –20°C. After thawing, cytosmears were fixed for 30 s at room temperature in a buffered formol/acetone solution, followed by a preincubation with 5% non-fat dry milk in PBS for 10 min. The first antibody, either the anti-5T2 or anti-5T33 (3 μg $ml^{-1}$), was incubated for 30 min followed by a second preincubation with 5% decomplemented AB serum for 10 min. After rinsing, colloidal gold-labelled (5 nm) goat anti-mouse $IgG_1$ antibodies (Auroprobe LM, Amersham International, UK), at a dilution of 1:75, were incubated for 30 min followed by extensive rinsing in distilled water. Silver enhancement was subsequently performed with the Intense B1 Silver Enhancement kit (British Biocell International, Sanvertech, Cardiff, UK) for 15 min at 26°C (Iqbal et al, 1993). Cytospins were counterstained with May–Grünwald–Giemsa.

As to the assessment of phenotypic adhesion profile, anti-LFA 1α chain (clone M1714), anti-Mac-1 α-chain (clone M1/70), anti-VLA-4 α-chain (clone R1-2), anti-VLA-5 α-chain (clone 5H10-27), anti-VLA β-chain (clone 9EG7) and anti-CD44 (clone 1M7) (all antibodies from Pharmingen, San Diego, CA, USA) were used at a dilution of 1:100 as primary antibody and gold-labelled goat anti-rat Ab (Auroprobe LM) were used as second step. As negative control, irrelevant antibodies of the same isotype were used as first step.

### Immunohistochemistry and PCNA staining

Frozen tissue sections from all organs were labelled with the 5T2 or the 5T33 specific anti-idiotype antibodies. For the visualization of the labelling we used the Histomouse kit (Zymed, Sanbio, Uden, The Netherlands). Briefly, two blocking steps were followed by overnight incubation at 4°C with the biotinylated anti-5T2 or 5T33

© Cancer Research Campaign 1997

**Figure 6** Kinetic series of (**A**) 5T2 MM and (**B**) 5T33 MM. Three mice were evaluated at each point in time. The evolution of the serum paraprotein concentration (mean of three mice, standard deviation was less than 20% of the mean) was for both lines correlated with the occurrence of the MM cells in the tissues and for the 5T2 MM also with the development of osteolysis. Osteolysis was not consistently observed in the 5T33 MM-bearing mice. The tissue involvement was assessed by both morphology and immunohistochemistry. (BM, bone marrow; S, spleen; L, liver; LN, lymph nodes)

antibody (1 μg $ml^{-1}$), followed by rinsing and incubation with the streptavidin–peroxidase complex for 30 min at room temperature. Subsequently, a DAB reaction was performed followed by counterstaining with haematoxylin. Because of the strong paraprotein secretion, multiple washings between each step were needed to avoid background staining. As negative control, corresponding frozen tissue, obtained from tumour-free C57BL/KaLwRij mice, was used in addition to irrelevant antibodies of the same isotype (anti-idiotype MAb B1) on tumoral material.

For PCNA staining, cytosmears of isolated 5T2 and 5T33 MM samples were fixed for 10 min in 4% formol in PBS. After rinsing in PBS, endogenous peroxidase was blocked with 0.3% hydrogen peroxide in methanol, again followed by rinsing in distilled water and PBS. Normal goat serum (10% in 0.8% BSA in PBS) was added on the sections for 10 min followed by an overnight incubation with MAb to NCL-PCNA (clone PC10) (Novocastra Laboratories, Newcastle upon Tyne, UK) at a dilution of 1:50 in PBS at 4°C, while irrelevant antibodies of the same isotype were

**Table 1** Phenotypic adhesion profile as assessed by immunogold silver staining on cytosmears

| | 5T2 MM | 5T33 MM |
|---|---|---|
| CD11a (α-chain LFA-1) | 99.1 ± 0.7 | 99.8 ± 0.2 |
| CD11b (α-chain Mac 1) | 2.4 ± 2.5 | 1.2 ± 0.3 |
| CD29 (β-chain VLA) | 99.7 ± 0.3 | 99.6 ± 0.0 |
| CD44 (H-CAM) | 99.1 ± 0.2 | 99.7 ± 0.3 |
| CD49d (α-chain VLA-4) | 99.5 ± 0.5 | 99.6 ± 0.4 |
| CD49e (α-chain VLA-5) | 99.6 ± 0.1 | 99.9 ± 0.1 |

Five hundred MM cells were counted on each slide. Three independent experiments were performed. Data are presented as the means ± standard deviation. LFA-1, lymphocyte function antigen-1; VLA, very late activation antigen; H-CAM, homing-associated cell adhesion molecule used as controls. After extensive rinsing with PBS, anti-mouse $IgG_{2a}$-specific antibodies coupled to horseradish peroxidase (Southern Biotechnology Associates, Birmingham, UK) were added at a dilution of 1:100 for 30 min. After rinsing with PBS, peroxidase was visualized by a DAB reaction, followed by a brief counterstaining with May–Grünwald–Giemsa. For each 5T2 and 5T33 MM, 500 MM cells were counted in three independent experiments. Results are presented as the mean with standard deviation.

### Cell preparation for electron microscopy

Cells were spun to a pellet with a Beckman Microfuge (Analys, Namen, Belgium) in Eppendorf tubes in a solution of 2.5% glutaraldehyde. This was followed by 1-h incubation with 0.1% osmium tetroxide. Pellets were subsequently dehydrated in a graded ethanol series, infiltrated with propylene–oxide–Epon and embedded in Epon. Ultrathin sections were stained with uranyl acetate and lead citrate. They were subsequently examined by transmission electron microscopy (Zeiss TEM 109, Oberkochen, Germany).

### Radiography

Bone lesions were evaluated in mice by radiography dedicated for mammography. Briefly, mice were anaesthetized and a radiograph of pelvis and hind legs was performed (Vanderkerken et al, 1996). This method allowed the follow-up of bone lesions in the same mouse.

### RESULTS

### Histology

Thirty 5T2 MM and 30 5T33 MM inoculated mice and ten control mice were investigated for organ infiltration with MM cells 13 and 4 weeks after transplantation respectively.

For the 5T2 MM cells (Figures 2–4) all mice had bone marrow infiltration 13 weeks after inoculation whereas the spleen was invaded in only 46% of mice (Figure 4) with a focal infiltration and a 1.5-fold increase in weight. An infiltration of the liver was observed in only 7% of mice, whereas lymph nodes were invaded minimally in 25% of mice. No tumoral cells were observed in the peripheral blood.

The infiltration of the 5T2 MM cells in the bone marrow was uneven. At sites of bone involvement, myeloma cells showed a diffuse growth pattern. Although most myeloma cells had a plasmablastic morphology with extensive rough endoplasmic reticulum

 © Cancer Research Campaign 1997

**Figure 7** Radiograph of the femur of a 5T2 MM-positive animal showing numerous osteolytic lesions (arrows)

(Figure 5), some features of plasma cell differentiation, such as a slightly eccentrically located nucleus and a cartwheel chromatin pattern, could also be observed. With the Gomori stain no increase in reticulin fibres was observed. At sites of involvement, a decrease in the number of bone trabeculae could consistently be observed. The cortical bone was thinned compared with the control mice. The liver infiltration consisted of a small solitary aggregate composed of a few MM cells. An increase in liver weight was never observed. The lymph node infiltration was mainly localized in the sinuses and the interfollicular areas and was never accompanied by a periganglionic spread.

The organ distribution of myeloma cells was confirmed with immunohistochemical labelling on frozen sections using the MM anti-idiotype antibodies (Figure 3). The MM cells showed a cytoplasmic positivity with variable intensity. No positive cells could be observed in the tissues from the control mice.

All 5T33 MM-inoculated mice showed a bone marrow infiltration. The liver and spleen were involved in 82% and 96% of the mice respectively. This tissue infiltration was associated with a hepatomegaly (threefold increase in weight) and a splenomegaly (20- to 30-fold increase). At least one lymph node was infiltrated in 58% of tested mice (Figures 2–4). Heart, blood, lung, intestine, kidneys, thymus and testes remained tumour free. Histological study of all infiltrated organs has been performed. The myeloma cell population in the bone marrow was unevenly distributed. At sites of bone marrow involvement, the myeloma cell growth was diffuse. At sites of bone destruction, a local spread into the surrounding soft tissue could be observed. The MM cells had a blastic morphology with a vesicular nucleus and several distinct nucleoli and extensive rough endoplasmic reticulum (Figure 5). The cytoplasm was basophilic on the Giemsa staining. There was a marked polymorphism. Mitotic figures could easily be found. There was no increase in reticulin fibres on the Gomori stain of the bone marrow samples. A destruction of cortical bone and a decrease in bone trabeculae was observed at sites of invasion. In the liver the myeloma cells were located along the sinuses and the portal tracts. In the spleen all 5T33 MM cells were observed in the red pulp. Massively infiltrated lymph nodes showed a periganglionic spread.

PCNA staining illustrated the higher proliferative index of the 5T33 MM cells, when compared with the 5T2 MM cells: 55.6 ± 9.0 and 5.3 ± 1.1 of the MM cells (mean ± standard deviation) were positive, for 5T33 and 5T2 MM cells respectively.

### Kinetics

In the kinetic series of the 5T2 MM (Figure 6A), isolated idiotype-positive cells were observed by immunohistochemistry from week 9 in some bone marrow and spleen samples. From week 10, all bone marrow samples and half of the spleen samples contained idiotype-positive cells, which was also confirmed by morphology. Lymph nodes were infiltrated from week 13. Significant paraprotein was detected in the serum by proteinelectrophoresis from week 9 and the concentration augmented during the course of the disease. Positivity in ELISA could be detected from week 4. Minimal osteolytic lesions could be observed from week 10, all evolving to numerous small cavities (Figure 7) and eventually resulting in bone fractures at the terminal stage of the disease (from week 20). At this last stage, no other organs than bone marrow, spleen and liver were histologically involved by the 5T2 MM cells.

For 5T33 MM (Figure 6B), both immunohistochemistry with the anti-5T33 MM idiotype MAb and the morphology on paraffin sections revealed the involvement of bone marrow, liver and spleen from week 2. At the same time, paraprotein could be detected in the serum by protein electrophoresis. ELISA could demonstrate paraprotein in the serum from week 1. In the terminal stage of the disease (from week 6), paralysis of the hind legs is often observed. As in the 5T2 MM, no other organs were involved at this stage than those mentioned above. Osteolysis could be observed in some animals from week 4, but was not consistent for all.

### Phenotypic adhesion profile

Table 1 illustrates the phenotypic adhesion profile as assessed by immunogold silver staining on cytosmears. Both 5T2 and 5T33 MM cells isolated from the bone marrow were LFA-1 α-chain (CD11a) positive, Mac-1 α-chain (CD11b) negative, VLA-4 (CD49d–CD29) positive, VLA-5 (CD49e-CD29) positive. The 5T2 and 5T33 MM cells were recognized on the basis of their typical blastic morphology.

### DISCUSSION

The general purpose of this work was to study the homing and organ distribution and the phenotypic adhesion profile of experimental mouse multiple myeloma lines. The 5T2 and 5T33 MM lines that spontaneously occurred in C57BL/KaLwRij mice (Radl et al, 1979) and have since been propagated in vivo by intravenous

© Cancer Research Campaign 1997

transfer into young syngeneic recipients. Both lines share similar general properties with human MM: spontaneous origin, the development of the disease can be monitored by the serum paraprotein concentration and osteolysis occurs.

Homing of lymphocytes is generally defined as the selective recruitment of specific lymphocyte subsets, under strict microenvironmental control (Picker et al, 1994). The local microenvironment differentially regulates the adhesion molecule expression and thus controls the adhesion of specific lymphocyte subsets to cells or extracellular matrix proteins within a tissue. A multistep model of this homing has been proposed (Picker et al, 1994): an initial unstable adhesion to endothelial cells is then followed by an activation-dependent stable secondary adhesion. This can subsequently be followed by transendothelial migration into the tissues. Here, chemoattractants will regulate the migration and tertiary adhesion to tissue components such as fixed cells or extracellular matrix components. The potential requirements of the different sequential regulated steps generates a specificity that exceeds that of its component steps (Butcher and Picker, 1996).

To influence this homing and growth of MM cells through adhesion molecules involved in the secondary and tertiary adhesion, the phenotypic adhesion profile of the MM cells and a detailed study of all organs involved by these tumoral 5TMM cells is necessary. Thus, mice injected intravenously with myeloma cells were submitted to a serial killing experiment. When a clear-cut serum paraprotein concentration was observed in all mice, the organ distribution of the two 5T MM lines was analysed by a combination of histology and immunohistochemistry. For the latter, specific monoclonal anti-idiotype antibodies were developed for each line. FACS analysis, immunostainings on cytosmears and cryostat sections as well as ELISA demonstrated the specificity of these antibodies. All mice analysed for each line exhibited a homologous tissue pattern.

The growth of the 5T2 MM cells was more restricted to the bone marrow and the disease showed a less progressive evolution. Nearly half of the mice showed a limited infiltration of the spleen, whereas only a very low percentage of mice showed an infiltration in the liver. In contrast, Radl et al (1988) did not find a liver infiltration in the 5T2 MM-bearing mice.

The 'take' of the 5T33 MM cells was fast, and by the fourth week a massive infiltration of bone marrow, spleen and liver resulted in paralysis of hind legs, splenomegaly and hepatomegaly. Lymph nodes were infiltrated in most of the mice. A periganglionic spread and spread into the surrounding soft tissue at sites of bone destruction was frequently observed.

The fact that the spleen is involved in both MM lines is not surprising as in mice, in contrast to humans, the spleen retains its haematopoietic function after birth. The infiltration of 5T33 and, occasionally, 5T2 MM in the liver can also be explained by the fact that the liver is the major site of haemopoiesis not only during fetal life, but also in adults and the liver still retains an environment that can support haemopoiesis (Taniguchi et al, 1996). In some cases, when the function of the bone marrow is severely suppressed, as after accidental radiation exposure, an extramedullary haemopoiesis occurs in the liver.

We did not observe an infiltration in the thymus, in either 5T2 or 5T33 MM (by both morphology and immunohistochemistry with the anti-idiotype antibodies); this is in contrast to the data of Manning et al (1992), who could identify a small subpopulation of cytoplasmic $Ig_{2b}$ cells in the thymus of 5T33 MM-inoculated mice by indirect immunofluorescence.

When comparing the organ involvement of the two 5TMM lines, we should stress that the 5T2 MM cells are mainly located in the bone marrow and only a minor infiltration is observed in red pulp of the spleen and liver, whereas in the 5T33 MM the bone marrow infiltration is accompanied by a massive infiltration of liver and spleen in nearly all mice.

The higher proliferation index of the 5T33 MM cells, as illustrated by PCNA staining, confirmed their more aggressive growth when compared with the 5T2 MM cells. PCNA/cyclin is a highly conserved (between species) acidic nuclear protein that is synthesized in late $G_1$ and S phase and is present throughout the cell cycle except in the $G_0$ state. PCNA expression is therefore correlated with the cell cycle. Several authors have suggested this staining as an alternative for thymidine pulse labelling, especially in pre-existing fixed or frozen materials (Galand et al, 1989; Kawakita et al, 1992). We are aware that PCNA staining gives an overestimation of proliferation when compared with 'classical' thymidine incorporation because all cells, except those in $G_0$ state, are stained. However, the clear difference in staining between the two cell lines, 5T2 and 5T33 MM, confirms the more proliferative behaviour of the 5T33 MM line, which is in agreement with the observations of numerous mitotic figures and with their rapid 'take' time (even after injection of 20 times fewer cells).

The kinetic experiments performed for both lines clearly demonstrate a correlation between the occurrence of MM cells, the serum paraprotein content and, for the 5T2 MM, the development of osteolytic lesions. For both lines, all organs, i.e. bone marrow and spleen for 5T2 MM and bone marrow, spleen and liver for 5T33 MM, were infiltrated simultaneously and at the terminal stage of the disease no 'overflow' to other organs was observed, indicating their homing restriction to haematopoietic environments.

A striking feature of human MM is that the malignant plasma cells home to and proliferate in the bone marrow. Only in advanced stages of the disease are circulating MM cells observed in the peripheral blood. The interactions between MM cells and stromal cells and extracellular matrix proteins are mediated by cellular adhesion molecules. These molecules can be divided into four families: the Ig superfamily, the integrins, the selectins and the cadherins. Studies on human MM implied mainly the integrin family and CD44 (H-CAM). Integrins are known to be important in the dynamic regulation of adhesion and migration of lymphocytes. They consist of Ab heterodimers. β1 and β3 integrins mediate cell–matrix interactions, whereas the β2 integrin is responsible for cell–cell contact. β1 integrin was extensively studied in human MM. All MMs appeared to be α4β1 (VLA-4) positive (Van Riet and Van Camp, 1993; Pellat-Deceunynk et al, 1995*b*; Kawano, 1993). It was demonstrated that VLA-4–fibronectin interaction is necessary for the terminal differentiation of lg-secreting bone marrow cells (Roldan et al, 1992). The α5β1 (VLA-5) integrin is expressed on subpopulations of freshly isolated MM cells (Pellat-Deceunynk et al, 1995*b*; Kawano et al, 1993). Furthermore, the expression of VLA-5 is associated with more mature subpopulations. The expression of $\alpha_L$ integrin, LFA-1 (CD11a), is more controversial. Some authors have demonstrated that MM cells are LFA-1 negative (Van Riet and Van Camp, 1993), whereas others observed a correlation with LFA-1 expression in patients with fulminant disease (Ashmann et al, 1992). Furthermore, a correlation between VLA-5 and CD11a expression was observed in some patients (Kawano et al, 1993; Pellat-Deceunynk et al, 1995*b*). LFA-1–ICAM-1 interactions have been found to be responsible for homotypic cell aggregations of human MM in culture (Kawano et

 © Cancer Research Campaign 1997

al, 1991). Several authors have reported the absence of the CD11b marker on all myeloma cells studied (Uchiyama et al, 1992; Van Riet and Van Camp, 1993; Kim et al, 1994; Pellat-Deceunynk et al, 1995*a*). Mac-1 (CD11b-CD18) is mainly expressed on monocytes and granulocytes and is involved in the adhesion of these cells to endothelial cells and their localization at sites of inflammation.

CD44 has been linked to site-specific extravasation of lymphocytes into tissues, to site-directed homing of B and T lymphocytes and to binding to extracellular matrix proteins. Degrassi (1993) demonstrated that the interaction of CD44 with its ligand hyaluronate was responsible for part of the adhesion of plasmacytomas on stromal cell layers.

Our findings of phenotypic adhesion profile in the 5T2 MM, i.e. LFA-1, VLA-4, VLA-5, CD44 positivity and Mac-1 negativity, thus corresponds to the data obtained on freshly isolated human MM cells.

The 5T33 MM model may be representative of an aggressive human variant of MM (Bartl R et al, 1991) that is clinically characterized by a rapidly aggressive course with a mean survival of less than 6 months and a high incidence of hepatosplenomegaly and complications. This variant is histologically characterized by a packed marrow, a blastic cell type and a high mitotic rate.

In contrast, the 5T2 MM model more closely resembles the classical presentation of human MM in several aspects: the course of the disease is moderately progressive, the homing is more restricted to the bone marrow, some maturation towards the plasma cell can be observed and the development of osteolysis can consistently be observed during the course of the disease (Radl, 1985; Vanderkerken et al, 1996). Further, the in vitro growth is 'stroma dependent'. Preliminary experiments could demonstrate some growth of 5T2 MM in vitro when co-cultured with a stromal cell layer (consisting of bone marrow fibroblasts) (unpublished data). In contrast, 5T33 MM cells grow in a stroma-independent fashion (Degrassi et al, 1993).

Most artificial animal models use human MM cell lines injected into severe combined immunodeficient (SCID) mice (Huang et al, 1993; Alsina et al, 1995), chemically induced mouse plasmacytomas (Degrassi et al, 1993) or genetically modified hybridomas (Okada et al, 1995). As in human MM, it is expected that the interactions of mouse MM cells with the stromal cells and osteoclasts are based on a complex network of cytokines and adhesion molecules, inducing a paracrine proliferation of MM. The abovementioned experimental models might lack these interactions. In addition, a recent report (Pellat-Deceunynk et al, 1995*a*) stresses the importance of choice of human MM lines in the study in SCID mice. It appears that lymphoblastoid cell lines (LCL) are frequently being used. These lines are formed by immortalization of non-malignant B cells by Epstein–Barr virus (EBV) and present karyotypes completely different from those of genuine human MM lines which are EBV negative and which have chromosomal abnormalities identical to those of fresh tumour cells.

We conclude that the 5T2 MM very closely resembles the human disease and therefore is more suitable for the study of the biology of MM than most artificial models. Future work will focus on the mechanism of selective homing of the MM cells to the bone marrow. Studies on human co-cultures of MM cells with fibroblasts (Uchiyama et al, 1993; Lokhorst et al, 1994; Faid, 1996) have already demonstrated that interactions other than with the known adhesion molecules are involved in the adhesion and proliferation of MM cells. We will therefore also consider mechanisms like those involving cytoskeletal and/or motility changes.

### ACKNOWLEDGEMENTS

The authors thank Nicole Buelens for the histology and PCNA stainings, Pascal Verhavert for the immunohistochemical stainings, Joeri De Nayer for his help with the immunogold stainings of the cytospins and Manu Wyfels and Petra Roman for their help with the transplantation of the animals. This work was partially supported by BIOMED contract no. BMHI-CT93-1407, by OZR contract no. 1963321210 of the Free University Brussels, the Ministry of Science (GOA) and Vereniging voor Kankerbestrijding.

### REFERENCES

Alsina M, Boyce BF, Mundy GR and Roodman GD (1995a) An in vivo model of human multiple myeloma bone disease. *Stem Cells* **13**: 48–50

Alsina M, Boyce B, Devlin RD, Anderson JL, Craig F, Mundy GR and Roodman GD (1996) Development of an in vivo model of human multiple myeloma bone disease. *Blood* **4**: 1495–1501

Ashmann EJM, Lokhorst HM, Dekker AW, Bloem AC (1992) Lymphocyte-function-associated antigen-1 expression on plasma cells correlates with tumor growth in multiple myeloma. *Blood* **79**: 2068–2075

Bartl R, Frisch B, Diem H, Mündel M, Nagel D, Lamerz R, Fateh-Moghadam A (1991) Histologic, biochemical and clinical parameters for monitoring multiple myeloma. *Cancer* **6**: 2241–2250

Bataille R, Chappard D, Marcelli C, Dessauw P, Baldet P and Alexandre C (1989) Mechanisms of bone destruction in multiple myeloma: The importance of an unbalanced process in determining the severity of lytic bone disease. *J Clin Oncol*, **7**: 1909–1914

Brissinck J, Demanet C, Moser M, Oberdan L and Thielemans K (1991) Treatment of mice bearing $BCL_1$ lymphoma with bispecific antibodies. *J Immunol* **147**: 4019–4026

Butcher EC and Picker LJ (1996) Lymphocyte homing and homeostasis. *Science* **272**: 60–66

Degrassi A, Hilbert DM, Rudikoff S, Anderson AO, Potter M and Coon HG (1993) In vitro culture of primary plasmacytomas requires stromal cell feeder layers. *Proc Natl Acad Sci USA* **90**: 2060–2064

Faid L, Van Riet I, De Waele M, Facon T, Schots R, Lacor P and Van Camp B (1996) Adhesive interactions between tumour cells and bone marrow stromal elements in human multiple myeloma. *Eur J Haematol* **57**: 349–358

Galand P and Degraef C (1989) Cyclin/PCNA immunostaining as an alternative to tritiated thymidine pulse labelling for marking S phase cells in paraffin sections from animal and human tissues. *Cell Tissue Kinet* **22**: 383–392

Huang YW, Richardson JA, Tong AW, Zhang BQ, Stone MJ and Vitetta ES (1993) Disseminated growth of a human multiple myeloma cell line in mice with severe combined immunodefiency disease. *Cancer Res* **53**: 1392–1396

Iqbal A, Segers E, Renmans W, Jochmans K and De Waele M (1993) Immunophenotyping of leukemia and lymphoma cells with IGSS: comparison with APAAP and flow cytometry. *J Histotechnol* **16**: 263–270

Kawakita N, Seki S, Sakaguchi H, Yanai A, Kuroki T, Mizoguchi Y, Kobayashi K and Monna T (1992) Analysis of proliferating hepatocytes using a monoclonal antibody against proliferating cell nuclear antigen/cyclin in embedded tissues from various liver diseases fixed in formaldehyde. *Am J Pathol* **140**: 513–520

Kawano MM, Huang N, Tanaka H, Ishikawa H, Sakai A, Tanabe O, Nobuyoshi M and Kuramoto A (1991) Homotypic cell aggregations of human myeloma cells with ICAM-1 and LFA-1 molecules. *Br J Haematol* **79**: 583–588

Kawano MM, Huang N, Harada H, Harada Y, Sakai A, Tanaka H, Iwato K and Kuramoto A (1993) Identification of immature and mature myeloma cells in the bone marrow of human myelomas. *Blood* **82**: 564–570

Kim I, Uchiyama H, Chauchan D and Anderson KC (1994) Cell surface expression and functional significance of adhesion molecules on human myeloma-derived cell lines. *Br J Haematol* **87**: 483–493

Lokhorst HM, Lamme T, de Smet M, Klein S, de Weger RA, van Oers R and Bloem AC (1994) Primary tumor cells of myeloma patients induce interleukin-6 secretion in long-term bone marrow cultures. *Blood* **84**: 2269–2277

Manning LS, Berger JD, O'Donoghue HL, Sheridan GN, Claringbold PG and Harvey Turner J (1992) A model of multiple myeloma: culture of 5T33 murine myeloma cells and evaluation of tumorigenicity in the C57BL/KaLwRij mouse. *Br J Cancer* **66**: 1088–1093

Okada T and Hawley RG (1995) Adhesion molecules involved in the binding of murine myeloma cells to bone marrow stromal elements. *Int J Cancer* **63**: 823–830

© Cancer Research Campaign 1997 

Pellat-Deceunynk C, Amiot M, Bataille R, Van Riet I, Van Camp B, Omede P and Boccadoro M (1995*a*) Human myeloma cell lines as a tool for studying the biology of multiple myeloma: a reappraisal 18 years after. *Blood* **86**: 4001–4002

Pellat-Deceunynk C, Barillé S, Puthier D, Rapp MJ, Harousseau JL, Bataille R and Amiot M (1995*b*) Adhesion molecules on human myeloma cells: significant changes in expression related to malignancy, tumor spreading and immortalization. *Cancer Res* **55**: 3647–3653

Picker E (1994) Control of lymphocyte homing. *Curr Opin Immunol* **6**: 394–406

Radl J, De Glopper E, Schuit HR and Zurcher C (1979) Idiopathic paraproteinemia. II. Transplantation of the paraprotein-producing clone from old to young C57BL/KaLwRij. *J Immunol* **122**: 609–613

Radl J, Croese JW, Zurcher C, van den Enden-Vieveen MHM, Brondijk RJ, Kazil M, Haaijman JJ, Reitsma PH and Bijvoet OLM (1985) Influence of treatment with APD-biphosphonate on the bone lesions in the mouse 5T2 multiple myeloma. *Cancer* **55**: 1030–1040

Radl J, Croese J, Zurcher C, van den Enden-Vieveen MHM and de Leeuw AM (1988) Multiple myeloma: animal model of human disease. *Am J Pathol* **132**: 593–597

Roldan E, Garcia-Pardo A and Brieva JA (1992) VLA-4-fibronectin interaction is required for the terminal differentiation of human bone marrow cells capable of spontaneous and high rate immunoglobulin secretion. *J Exp Med* **175**: 1739–1747

Taniguchi H, Toyoshima T, Fukao K and Nakauchi H (1996) Presence of hematopoietic stem cells in the adult liver. *Nature Med* **2**: 198–203

Uchiyama H, Barut BA, Chauchan D, Cannistra SA and Anderson KC (1992) Characterization of adhesion molecules on human myeloma cell lines. *Blood* **80**: 2306–2314

Uchiyama H, Barut BA, Mohrbacher AF, Chauchan D and Anderson KC (1993) Adhesion of human myeloma-derived cell lines to bone marrow stromal cells stimulates interleukin-6 secretion. *Blood* **82**: 3712–3720

Van Riet I and Van Camp B (1993) The involvement of adhesion molecules in the biology of multiple myeloma. *Leuk Lymph* **9**: 441–452

Vanderkerken K, Goes E, De Raeve H, Radl J and Van Camp B (1996) Follow-up of bone lesions in an experimental multiple myeloma mouse model: description of an in vivo technique using radiography dedicated for mammography. *Br J Cancer* **73**: 1463–1465

 © Cancer Research Campaign 1997